# Difference in the Breast Milk Proteome between Allergic and Non-Allergic Mothers

**DOI:** 10.1371/journal.pone.0122234

**Published:** 2015-03-23

**Authors:** Kasper A. Hettinga, Fabiola M. Reina, Sjef Boeren, Lina Zhang, Gerard H. Koppelman, Dirkje S. Postma, Jacques J. M. Vervoort, Alet H. Wijga

**Affiliations:** 1 Dairy Science and Technology, Food Quality and Design group, Wageningen University, Wageningen, The Netherlands; 2 Laboratory of Biochemistry, Wageningen University, Wageningen, The Netherlands; 3 University Medical Center Groningen, Department of Pediatric Pulmonology and Pediatric Allergology, Beatrix Children’s Hospital, GRIAC Research Institute, University of Groningen, Groningen, the Netherlands; 4 University Medical Center Groningen, Department of Pulmonology, GRIAC research institute, University of Groningen, Groningen, The Netherlands; 5 Center for Nutrition, Prevention and Health Services, National Institute of Public Health and the Environment, Bilthoven, The Netherlands; Indiana University, UNITED STATES

## Abstract

**Background:**

Breastfeeding has been linked to a reduction in the prevalence of allergy and asthma. However, studies on this relationship vary in outcome, which may partly be related to differences in breast milk composition. In particular breast milk composition may differ between allergic and non-allergic mothers. Important components that may be involved are breast milk proteins, as these are known to regulate immune development in the newborn. The objective of this study was therefore to explore differences in the proteins of breast milk from 20 allergic and non-allergic mothers. The results from this comparison may then be used to generate hypotheses on proteins associated with allergy in their offspring.

**Methods:**

Milk samples from allergic and non-allergic mothers were obtained from the PIAMA project, a prospective birth cohort study on incidence, risk factors, and prevention of asthma and inhalant allergy. Non-targeted proteomics technology, based on liquid chromatography and mass spectrometry, was used to compare breast milk from allergic and non-allergic mothers.

**Results:**

Nineteen proteins, out of a total of 364 proteins identified in both groups, differed significantly in concentration between the breast milk of allergic and non-allergic mothers. Protease inhibitors and apolipoproteins were present in much higher concentrations in breast milk of allergic than non-allergic mothers. These proteins have been suggested to be linked to allergy and asthma.

**Conclusions:**

The non-targeted milk proteomic analysis employed has provided new targets for future studies on the relation between breast milk composition and allergy.

## Introduction

Because of a range of health benefits, the WHO recommends to breastfeed babies exclusively for the first 6 months of life [1]. In addition to nutritional benefits, it is important for gut maturation and modulation of inflammatory response [2–4]. Furthermore, breast milk may directly modulate the newborn’s immune system, amongst others by delivering antibodies specific for microbes and other antigens present in the mother’s indoor and outdoor environment [2, 4, 5].

Breastfeeding has been linked to a decrease [3, 5] as well as increase [6] in the occurrence of allergy and asthma. A recent review on the relation between breastfeeding and allergy concluded that there are shortcomings in the current evidence for a clear relationship, as studies showed a wide variety in results [7]. One of the hypotheses put forward in this review is that the variation in composition of breast milk may be related to the lack of a clear relationship between breastfeeding and allergy. The same uncertainty can be seen when the allergy status of the mother is taken into account. Some studies showed a more pronounced protection with a positive family history of asthma or atopy [8, 9]. On the other hand, it has been reported that breast-fed children had higher immunoglobulin E (IgE) levels compared with never breast-fed children, if their mothers had high IgE levels [10]. This controversy may in part be due to individual variations in the levels of immunological components in breast milk of allergic and of non-allergic mothers. Different components, including leukocytes, cytokines, immunoglobulins and fatty acids, have all been suggested as potential candidates for this effect [11]. For example, IgG–antigen immune complexes have been linked to allergy and asthma prevention [4], although other milk proteins, including α-lactalbumin and β-lactoglobulin, have also previously been linked to a reduction in prevalence of allergy and asthma [12]. In addition, cytokines in milk, including transforming growth factor-β (TGF-β) and interleukin-10 (IL-10), have previously been linked to tolerance induction [4].

Previous research aiming to find components in breast milk of allergic mothers that could explain the reduction in allergy in their offspring has focused on specific compounds thought to affect allergy or tolerance induction. To study these components, targeted techniques have been used. With recent developments in the field of non-targeted proteomics, it has become possible to screen many hundreds of proteins simultaneously instead of having to target specific preselected proteins.

In this paper, we report the use of non-targeted proteomics to study differences in the proteome of breast milk from allergic and non-allergic mothers.

## Methods

**Fig 1:**
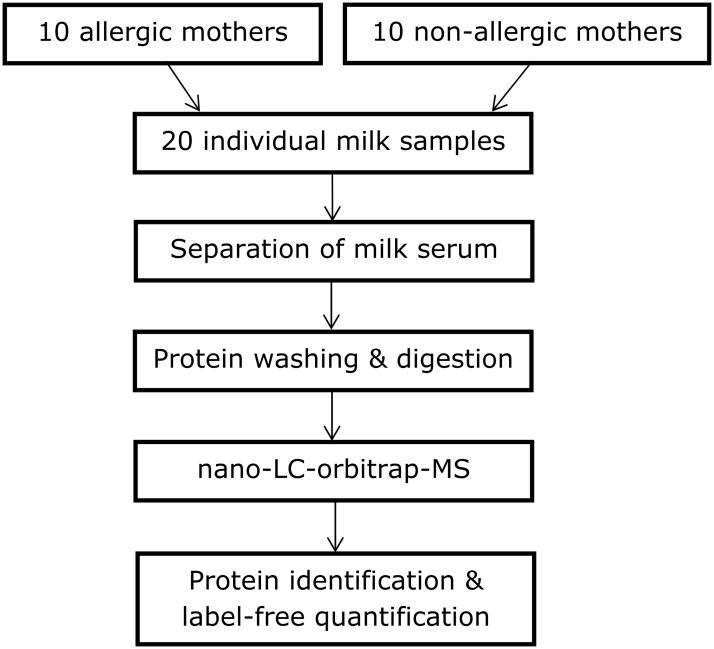
Overview of experimental procedure.

The procedure followed during the experimental phase is detailed below. A general flow diagram of the process is shown in Fig. 1.

### Milk samples

Samples analysed were obtained from the PIAMA project, which is a prospective birth cohort study on incidence, risk factors, and prevention of asthma and inhalant allergy [13].

At the time of recruitment, the pregnant mothers filled in a validated screening questionnaire on allergy [14]. Mothers reporting at least one of the following: (a history of) asthma, current hay fever, current allergy for pets, or current allergy for house dust or house dust mite were defined as ‘allergic’ and mothers reporting that they had none of these complaints were defined as ‘non-allergic’. Blood samples were obtained from a sub-group of the mothers with self-reported asthma or allergies and their allergic status was confirmed by tests for specific IgE against house dust mite. Only mothers with confirmed house dust mite (HDM) allergy were selected in the allergic group. HDM allergy was chosen, given that HDM is one of the most common allergens [8] and exposure to this allergen cannot be prevented.

Breast milk samples were taken when the infants were 2 to 4 months old. For this study, 20 samples were analysed, 10 samples from mothers with confirmed HDM allergy with high HDM allergen exposure, and 10 from mothers who reported no asthma or allergies, with a similar HDM allergen exposure. This number is based on a power calculation, aiming at picking up a 5-fold difference, a standard deviation of the same size, an alpha of 0.05, and beta of 0.20. The milk samples were collected in small plastic cups, either by manual pressure or by using a breast pump. The samples were stored at -80°C. Details on sample collection (time and mode of collection) can be found in S1 Table.

### Ethics Statement

This research was performed in accordance with the ethical principles for medical research involving human subjects outlined in the Declaration of Helsinki. The study protocol was approved by the Medical Ethics Committees of the participating institutes (Rotterdam MEC 132.636/1994/39 and 137.326/1994/130; Groningen MEC 94/08/92; Utrecht, MEC-TNO oordeel 95/50). All parents gave written informed consent.

### Removal of fat and casein

As the presence of fat and casein disturbs the proteomics analysis, these components were removed from milk, to obtain a transparent liquid called milk serum. Samples were first thawed at room temperature and then approx. 3mL of each was ultracentrifuged for 90min at 100,000g and 30°C, in a Beckman L-60 ultracentrifuge to separate milk serum.

### Protein quantification

Prior to preparing the samples for analysis, their protein content was measured using a BCA Protein Assay kit (Thermo, San Jose, CA, USA), to ensure that the same amount of protein (10 μg) was loaded onto each filter.

### Sample preparation for proteome analysis

The milk serum samples were prepared for LC/MSMS analysis according to the procedure described by Lu et al. [15].

First, 20μL of serum was diluted with 180μL of a solution of 100mM Tris/HCl (pH 8) containing 4% SDS and 0.1M DTT and then heated for 10min at 95°C. Afterwards, 10μL of each sample was loaded onto a Pall 3K omega filter and centrifuged at 20,000g for 1 min. Then, 100μL of a solution of 8M urea in 100mM Tris/HCl (solution A) was added and the device was centrifuged for 30 min. Next, 100μL of 0.05M iodoacetamide in solution A was added, followed by centrifugation for 1 min and incubation at room temperature for 30 min, and then another 30min of centrifugation. After that, each filter was washed with 110, 120 and 130μL respectively of solution A, and centrifuged for 30 min after each washing step. Finally, 140μL of 0.05M NH_4_HCO_3_ (solution B) was added, followed by centrifugation for 30min. The filter was then transferred to a clean low-binding microcentrifuge tube, and 1μL sequencing-grade trypsin (Roche, Germany) in 100μL solution B was added to each sample. The filters were incubated overnight at room temperature and then centrifuged for 30 min. Finally, 3.5μL of 10% TFA in water was added to each tube to reach a pH of 2–4.

### LC-MS/MS

Samples were analysed with LC-MS/MS by the department of Biochemistry at Wageningen University. Samples were analysed by injecting 18μL of sample over a 0.10*32mm Prontosil 300-3-C18H (Bischoff, Germany) pre-concentration column (prepared in house), at a maximum pressure of 270bar. Peptides were eluted from the pre-concentration column onto a 0.10*200mm Prontosil 300-3-C18H analytical column, with an acetonitrile gradient at a flow of 0.5μL/min. The gradient consisted of an increase from 9% to 21% acetonitrile in water with 1mL/L formic acid in 100min, followed by an increase in the percentage acetonitrile to 34% (with 68% water and 1mL/L formic acid) in 26 min, which was increased further to 50% acetonitrile in 3 min. Between the pre-concentration and analytical column, an electrospray potential of 3.5kV was applied directly to the eluent via a solid 0.5mm platina electrode fitted into a P777 Upchurch microcross. Full scan positive mode FTMS spectra were measured between m/z 380 and 1400 on a LTQ-Orbitrap (Thermo electron, San Jose, CA, USA). MSMS scans of the four most abundant multiply-charged peaks in the FTMS scan were recorded in data-dependent mode in the linear trap (MSMS threshold = 5,000).

### Peptide and protein identification

Each run with all MSMS spectra obtained was analysed with MaxQuant v.1.2.2.5 as described before [16]. Methionine oxidations, acetylation of protein N termini, and de-amidation of N and Q were specified as variable modifications. Carboxamidomethylation of cysteines was set as a fixed modification (enzyme trypsin, 2 missed cleavages, peptide tolerance 6 ppm, fragment ions tolerance 0.5 Da). The human Uniprot database (downloaded March 2012), as well as a set of protein sequences of common contaminants, was used. In addition, MaxQuant created a decoy database consisting of reversed sequences to calculate the false discovery rate (FDR). The maximum FDR was set to 0.01 on peptide and protein level. Minimum required peptide length was six amino acids. Finally, proteins were identified based on minimally 2 distinct peptides of which at least one should be unique and one should be unmodified. All known contaminants (i.e. keratins, trypsin), and proteins detected in less than half of the samples, were removed from the set of proteins identified. The GO-annotation of these proteins was determined using DAVID GO [17].

### Full proteome quantification

The quantification of the full proteome is based on the extracted ion current and is taking the whole three-dimensional isotope pattern into account, using peak intensities of all measured isotopes [18]. At least two quantitation events were required for a quantifiable protein. If a protein was not detected in a specific sample, the value was set to 10^3^ (minimum detection level). MaxQuant was used with two different algorithms for quantification, IBAQ and LFQ [19]. Intensity based absolute quantification (IBAQ) estimates the absolute amount of a protein as the sum of the intensities of all peptides, divided by the number of tryptic peptides that can theoretically be generated. Label-free quantification (LFQ) is an algorithm that calculates relative protein amounts, by using several layers of normalization. These normalization steps make LFQ better than IBAQ for comparison between samples, whereas IBAQ is more suitable as an indicator for absolute concentration. Proteins had to have at least three valid LFQ intensities in either the allergic or non-allergic group in the final results.

### Statistical analysis

Perseus software v.1.2.0.16 was used to test for significant differences between groups. The ratio between the concentration found in allergic mothers and non-allergic mothers was calculated as the difference (on ^10^log scale) of LFQ intensities. Differences between groups were then statistically tested using two-tailed t-test, correcting for multiple testing using permutation-based FDR [20].

## Results

Table 1 shows that the demographic characteristics of the allergic and non-allergic mothers were very similar. Also parameters for dietary intake did not differ significantly between the mothers (data not shown).

**Table 1:** Overview of the main characteristics of the allergic and non-allergic mothers (either mean +/- standard deviation or fraction).

	Allergic mothers (n = 10)	Non-allergic mothers (n = 10)
Age mother (years)	30.3 +/- 2.9	31.4 +/- 3.8
Age baby at breast milk sampling (days)	101 +/- 17	101 +/- 16
Gestational age (weeks)	40.2 +/- 1.1	40.6 +/- 0.8
Gestational or non-gestational maternal diabetes (yes/no/data not available)	0/5/5	0/7/3
Education level mother (low/medium/high)	3/4/3	3/3/3
Male babies	7	7
Atopic father	3	4
Smoking during pregnancy	1	0
Multiparity	5	7
Pets during pregnancy	4	4

A total of 364 proteins were identified and quantified in all human milk samples together. Of these proteins, 357 were found in the the milk of allergic mothers, and 355 in the milk of non-allergic mothers, with an overlap of 348 proteins. Only 9 proteins were uniquely found in the milk of allergic mothers and 7 proteins were uniquely found in the milk of non-allergic mothers (for details, see S2 Table). Below, the qualitative and quantitative differences in the proteome are presented.

### Qualitative differences in the proteome

In order to examine the function of the proteins, annotation according to gene ontology (GO) was performed using the online tool DAVID [17], as shown in Table 2. About one-third of all proteins have a GO annotation that is related to the immune system (including response to wounding, inflammatory response, and apoptosis).

**Table 2:** Overview of the most abundant GO annotation cluster (biological process) of the proteome of milk samples from allergic and non-allergic mothers.

GO annotation cluster	Protein count	
	Non-allergic	Allergic
Response to wounding	53	52
Carbohydrate catabolic process	29	28
Homeostasis	20	20
Coenzyme metabolic process	21	20
Cellular carbohydrate biosynthetic process	14	13
Regulation of apoptosis	45	44
Response to extracellular stimulus (nutrients)	17	15
Inflammatory response	35	36
Hydrogen peroxide metabolic process	16	15
Response to organic substance	44	42

### Quantitative differences in the proteome

**Fig 2:**
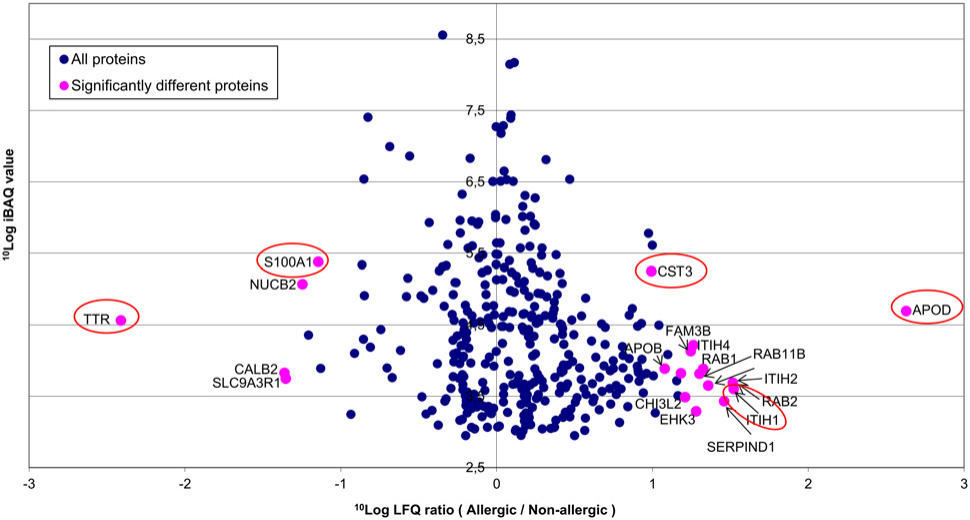
Overview of the differences in the proteome of milk from allergic and non-allergic mothers. Pink dots mark proteins that show significantly different concentrations between allergic and non-allergic mothers” while proteins marked with blue dots were not statistically different. Abbreviations are gene codes: TTR: Transthyretin; S100A1: S100 calcium-binding protein A1; NUCB2: Nucleobindin-2; CALB2: Calretinin; SLC9A3R1: Sodium-hydrogen exchanger regulatory factor 1; CST3: Cystatin-3; APOB: Apolipoprotein B; APOD: Apolipoprotein D; FAM3B: Pancreatic-derived factor; ITIH1: Inter alpha-trypsin inhibitor, heavy chain 1; ITIH2: Inter alpha-trypsin inhibitor, heavy chain 2; ITIH4: Inter alpha-trypsin inhibitor, heavy chain 4; RAB1: Ras-related protein Rab-1A; RAB1: Ras-related protein Rab-1A; RAB2: Ras-related protein Rab-2; RAB11B: Ras-related protein Rab-11B; SERPIND1: Heparin cofactor 2; EHK3: Ephrin type-A receptor 7 CHI3L2: Chitinase-3-like protein 2; Red encircled proteins are shown in more detail in Fig. 3.

First, we compared the differences in the proteome between allergic and non-allergic mothers. Fig. 2 shows the geometric mean ^10^log-ratio of the protein LFQ values of the milk of allergic mothers over that of non-allergic mothers. This figure shows the over-expressed proteins in allergic mothers on the right, and those under-expressed on the left. A total of 19 proteins were significantly different between allergic and non-allergic mothers (for details, see S2 Table). None of the proteins that were uniquely found in the allergic or non-allergic mothers were among the significantly different proteins. This is due to the fact that all proteins uniquely found in either group were very close to the detection limit, and that missing values were imputed with the detection limit for the statistical analysis.

**Fig 3:**
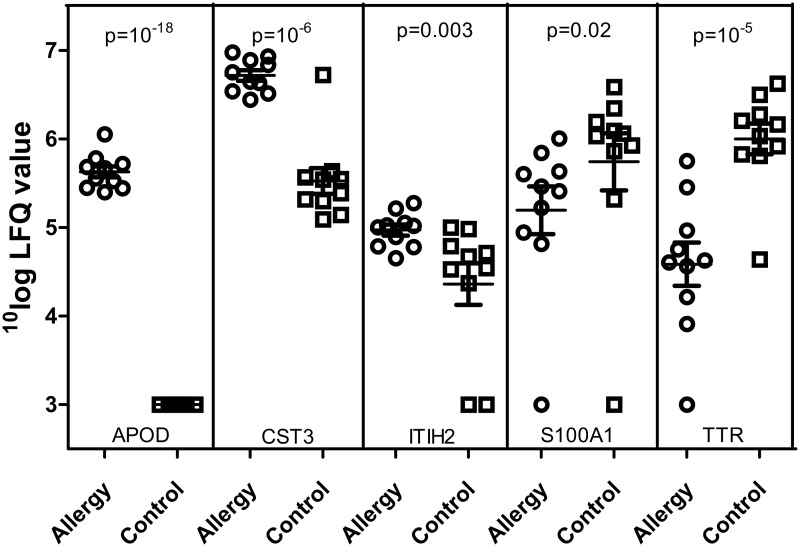
^10^log label-free quantification (LFQ) values for five proteins in individual samples from allergic and non-allergic (control) mothers. Proteins shown are APOD: Apolipoprotein D; CST3: Cystatin-C; ITIH2: Inter-alpha-trypsin inhibitor heavy chain H2; S100A1: S100 calcium-binding protein A1; TTR: Transthyretin. p-values are FDR-corrected p-values for t-test by Perseus.

We visualized whether these significant differences in protein intensity between the two groups of mothers were due to a few outliers or consistent differences between the groups, by plotting the data of individual mothers for five proteins. The five proteins were chosen to have relatively abundant proteins, representing two interesting biological functions (protease inhibitors and transport proteins), with large differences between the two groups. Additionally, we present two proteins that have higher and lower concentrations in allergic mothers compared to non-allergic mothers. Fig. 3 shows that for these five proteins, statistical differences were due to systematic differences between groups and not due to outliers.

## Discussion

### Overview of the breast milk proteome

Our proteomic study in breast milk of allergic and non-allergic lactating mothers shows that their proteomes are almost the same. This is in line with our observation that the proportion of proteins involved in the same biological processes is similar for the milk samples of allergic and non-allergic mothers (Table 2). In the statistical comparison, none of the 16 proteins that was uniquely identified in one of the groups of mothers was found to differ significantly. This can be explained by the fact that the concentrations of these proteins in the group in which the protein was present were often low and close to the detection limit, and missing values were imputed with the detection limit to enable statistical comparison. Of interest, the quantitative analysis of the proteome showed that the concentrations of several proteins were significantly different between the breastmilk of allergic and non-allergic mothers (Fig. 3). These results thus indicate that the difference between breast milk from allergic and non-allergic mothers lies mainly in the concentration of the proteins and not their absence or presence.

### Differences in the milk proteome between allergic and non-allergic mothers

Fig. 3, presenting data on individual mothers of five proteins, shows that there is considerable variation between individual mothers. We speculate that this large variation in milk proteome between individual mothers may be related to the large variation of results in studies on the relation between breastfeeding and asthma/allergy, as previously hypothesized by Matheson et al. [7]. Some cytokines previously related to tolerance induction (e.g. TGF-β and IL-10) were not found with our proteomics approach. This is to be expected given the very low concentration of these components.

The extent of the variation in the five proteins shown in Fig. 3 was similar to that seen for the other proteins within the entire proteome (see S2 Table for intensity and standard deviations of all proteins). However, for the above five proteins, the difference between mothers within one group is smaller than the difference between allergic and non-allergic mothers. For example, LFQ values for cystatin-C (a protease inhibitor) are consistently higher in allergic mothers than in non-allergic mothers. Based on these data of individual mothers, we conclude that the significant differences between groups are based on systematic differences between these groups, and not due to single outlying data points.

When looking more closely at the biological function of the 19 proteins that are significantly different between the two groups of mothers, one group of proteins is substantially different, i.e. the protease inhibitors. Cystatin C, several inter-alpha-trypsin inhibitors, and serine-protease inhibitors (SERPINS) are all present in higher concentrations in breast milk of allergic mothers. This seems remarkable, knowing that the major HDM allergen, Der p 1, is itself a protease and this proteolytic activity has been linked to the mechanisms of the allergic response [4]. Der p 1 is also known to degrade antiprotease-based lung defences such as mucosal α1-antitrypsin, which protects the respiratory mucosa against serine proteases [21]. In addition, it was previously shown that serine proteases and SERPINs are involved in the maintenance of the epithelial barrier of the skin and airways, and that an imbalance of protease-protease inhibitors allows easier penetration of allergens [22, 23]. For example, reduced cystatin secretion, a protease inhibitor we also detected in breast milk, by epithelial cells has been linked to easier penetration of Der p 1 through the epidermal barrier [24]. Because this proteolytic activity is considered relevant to the pathogenesis of asthma and allergy, protease inhibitors are being investigated as potential therapeutics for these diseases [23]. Protease inhibitors have previously been detected in stool of breastfed infants, indicating that these proteins are not fully digested, and may thus be active in the gastrointestinal tract [25]. In addition to a direct effect, protease inhibitors may also function through protection of other immune proteins against digestion [26, 27]. In summary, our results, combined with data from the literature indicate that protease inhibitors naturally present in breast milk may be an interesting group of proteins for further research and study of their potential beneficial effect. A useful next step would for example be to determine the concentration of protease inhibitors in a larger number of mothers in which the allergy status of their offspring is known. Unfortunately this was not possible in our study. However, this would allow assessing whether protease inhibitors in breast milk are associated with a reduction in the development of asthma and/or allergy in the offspring.

Next to the protease inhibitors, other milk proteins were found to be differentially expressed between allergic and non-allergic mothers. For example, apolipoproteins were found to be over-expressed in the milk of allergic mothers. Like protease inhibitors, apolipoproteins have been previously linked to asthma and atopy in children [28]; and more recently, their mimetic peptides have emerged as a potential new treatment for asthma [29]. Furthermore, our study provides also novel proteins that are different between allergic and non-allergic mothers, like transthyretin, which is carrier for the thyroid hormone thyroxine, and is also involved in retinol transport. The function of this protein, and other proteins that differed between allergic and non-allergic mothers, in relation to allergy or breast milk is unknown.

In conclusion, using non-targeted proteomics we identified 19 proteins that significantly differ between breast milk of allergic and non-allergic mothers. Protease inhibitors were the largest group of proteins that were upregulated in the milk of allergic mothers. If these results can be confirmed in future studies, this would show that unbiased milk proteomics analysis can provide new targets for studies on the relation between allergy and breast milk composition.

## Supporting Information

S1 Table Time and mode of breast milk collection. (XLSX)

S2 Table Overview of all identified protein, including signal intensities (^10^log LFQ values, ^10^log IBAQ values), including averages and standard deviation. Proteins present in only allergic or non-allergic mothers, and proteins that differ significantly between groups are indicated by an X in the respective three columns.
(XLSX)
